# Radiation-induced skin reactions: oxidative damage mechanism and antioxidant protection

**DOI:** 10.3389/fcell.2024.1480571

**Published:** 2024-10-09

**Authors:** Chuchu Liu, Jinlong Wei, Xuanzhong Wang, Qin Zhao, Jincai Lv, Zining Tan, Ying Xin, Xin Jiang

**Affiliations:** ^1^ Jilin Provincial Key Laboratory of Radiation Oncology and Therapy, The First Hospital of Jilin University and College of Basic Medical Science, Jilin University, Changchun, China; ^2^ Department of Radiation Oncology, The First Hospital of Jilin University, Changchun, China; ^3^ NHC Key Laboratory of Radiobiology, School of Public Health of Jilin University, Changchun, China; ^4^ Key Laboratory of Pathobiology, Ministry of Education and College of Basic Medical Science, Jilin University, Changchun, China

**Keywords:** radiation-induced skin reactions, mechanism, oxidative stress, treatment, antioxidant

## Abstract

According to official statistics, cancer remains the main reason of death and over 50% of patients with cancer receive radiotherapy. However, adverse consequences after radiation exposure like radiation-induced skin reactions (RISR) have negative or even fatal impact on patients’ quality of life (QoL). In this review we summarize the mechanisms and managements of RISRs, a process that involve a variety of extracellular and intracellular signals, among which oxidative stress (OS) are now commonly believed to be the initial part of the occurrence of all types of RISRs. As for the management of RISRs, traditional treatments have been widely used but without satisfying outcomes while some promising therapeutic strategies related to OS still need further researches. In the context we discuss how OS leads to the happening of RISRs of different types, hoping it can shed some light on the exploration of new countermeasures.

## 1 Introduction

Radiotherapy is widely used in clinic as the main means of controlling malignant tumors (Chandra et al., 2021). However, while the tumors are under effective control, the surrounding normal tissues and organs may be damaged to varying degrees due to the influence of radiation. Radiation-induced skin reactions (RISRs) is one of the main complications caused by radiation (Wei et al., 2019a). It usually occurs in the thin and wrinkled parts of the skin such as neck, armpit and groin. Radiation directly damages not only the skin but also its deep tissue cells, causing dryness, loss of elasticity, pigmentation, soft tissue fibrosis, capillary dilatation, and radiation dermatitis in irradiated areas (Yang et al., 2020), which often has a great impact on the course of radiotherapy, thus affecting the treatment effect of patients. Long-term sequelae will affect the QoL of patients (Bray et al., 2016).

**FIGURE 1:**
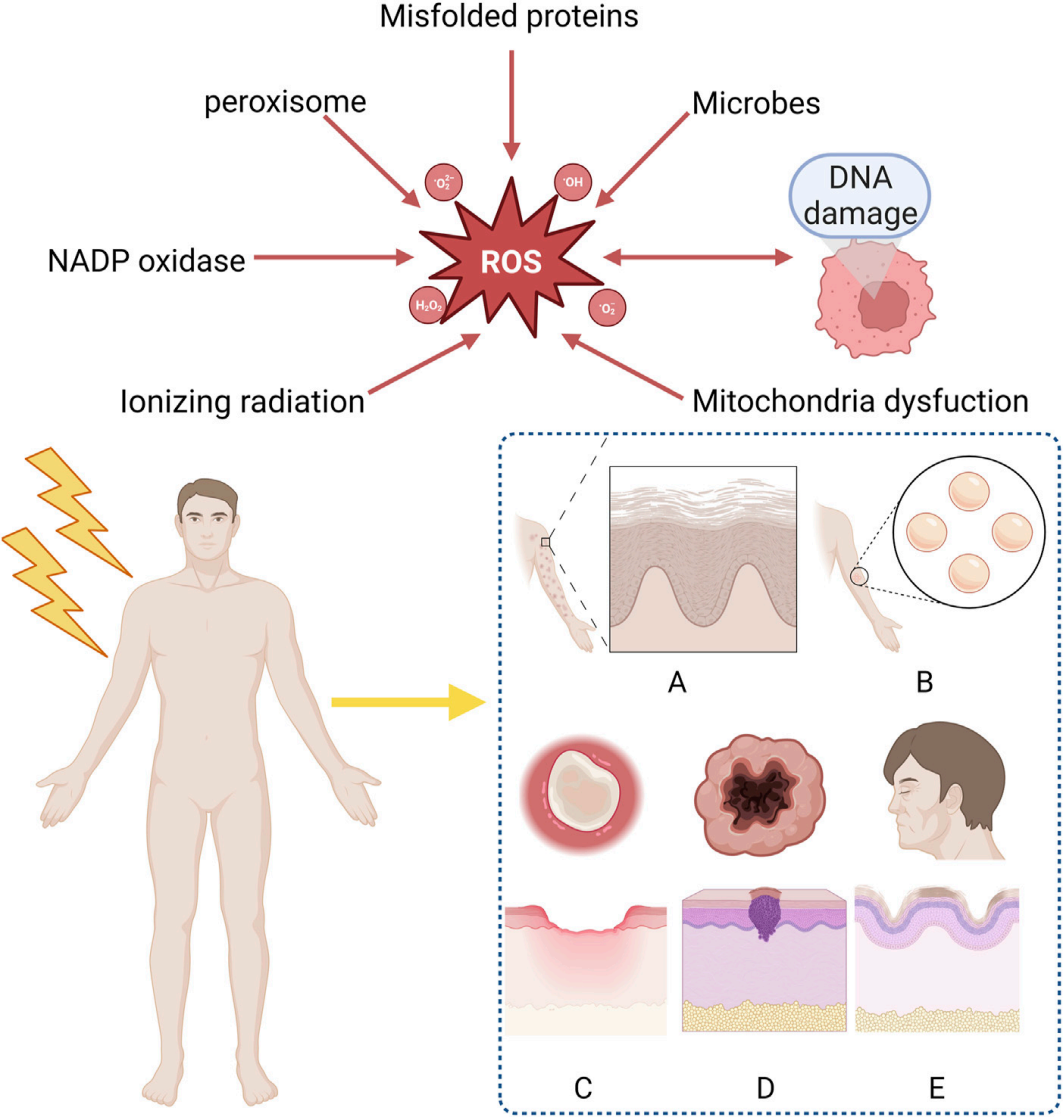
Clinical manifestation of radiation-induced skin reactions and sources of ROS. ROS mainly derive form ionizing radiation, mitochondria dysfunction, DNA damage, microbes, misfolded proteins, peroxisome, NADP oxidase and are considered as the reason why RISRs occur. Acute RISRs include acute erythema and bluster while chronic RISRs include chronic ulcers, fibrosis, skin aging and carcinogenesis. (RISRs: Radiation-induced skin reactions; ROS: Reactive oxygen species; NADP: Nicotinamide-adenine dinucleotide phosphate).

RISRs can be divided into acute RISRs and chronic RISRs according to the onset time. Acute RISRs are caused by one or multiple large doses of external radiation in a short period of time, including acute erythema and desquamation (Yang et al., 2020; Hegedus et al., 2017). Chronic RISRs are delayed adverse reactions that typically takes months to years to develop after exposure to ionizing radiation. Chronic RISRs include chronic ulcers, fibrosis, telangiectasia and skin carcinogenesis (Martin et al., 2016). This review aims at summarizing the oxidative stress mechanisms related to RISRs in order to search for potential targets of antioxidant treatments (Figure 1).

## 2 Oxidative stress mechanism

Oxidative damage is the major causes of RISR (Xue et al., 2021). OS is a situation when steady-state reactive oxygen species (ROS) concentration is transiently or chronically enhanced, disturbing cellular metabolism and its regulation and damaging cellular constituents (Lushchak, 2011). It is highly associated with the inflammation response (Mittal et al., 2014). ROS/RNS (reactive nitrogen species) includes superoxide, nitrogen oxide, hydrogen peroxide, hydroxyl radicals, peroxynitrite, etc. Based on the different types of RISRs, we analyzed the mechanisms of radiation-induced skin damage, fibrosis, skin carcinogenesis and skin aging respectively in the following paragraphs (Figure 1).

### 2.1 Radiation-induced skin damage

Radiation-induced skin damage, also known as acute forms of radiation dermatitis, occur within days to weeks, usually arise within 90 days. The most frequent symptoms are primary transient erythema, generalized erythema, skin dryness, hair loss, hyperpigmentation, dry desquamation, skin scaling and flaking and moist desquamation (Hegedus et al., 2017). From the molecular biology perspective, radiation causes skin injury by directly or indirectly damaging DNA and cellular structure. In other words, radiation-induced skin damage is the consequence of direct tissue injury in conjunction with local inflammatory reactions, including reduction and impairment of functional stem cells, endothelial cell changes, release of inflammatory factors, epidermal cell apoptosis and necrosis. IR can directly interact with DNA or indirectly through radiolysis of water. Exposure of DNA to IR can cause many different types of damage, and these damages can be roughly divided into DNA base damages, DNA single strand breaks (SSBs), and DNA double strand breaks (DSBs), with DSBs being considered the most dangerous type (Melia and Parsons, 2023). Even more tricky is that DNA damages caused by IR often does not occur singly, but rather generate clustered/complex DNA damage (CDD), containing two or more DNA lesions within one or two helical turns of the DNA. CDD is composed of various types of DNA damage, among which DSBs are the simplest form of CDD site (Wilkinson et al., 2023). Electron transfer or singlet molecular oxygen produced by radiation targets DNA base guanine, giving rise to 8-hydroxydeoxyguanosine (8-OHdG), a ubiquitous maker of OS, in the strand DNA (Ichihashi et al., 2003). After irradiation, the number of hydrogen molecule and hydroxyl (free radical) molecule in cells increases, causing the two-thirds of radiation-induced DNA damage (Sanche, 2009). The 2 hydroxyl molecules will recombine and form hydrogen peroxide, which is highly unstable, and then the hydrogen peroxide readily combines in the cell to form organic hydrogen peroxides molecule, which is stable. At this point, the radiation damage is “fixed” to the cells, resulting in the loss of an essential enzyme in the cell which could lead to cell death or a future mutation of the cell (Brown and Rzucidlo, 2011). Basically the extensive production of ROS and their fixation behavior on cells are the initial parts of almost all kinds of RISRs, gradually damaging human skin as time goes by.

**FIGURE 2:**
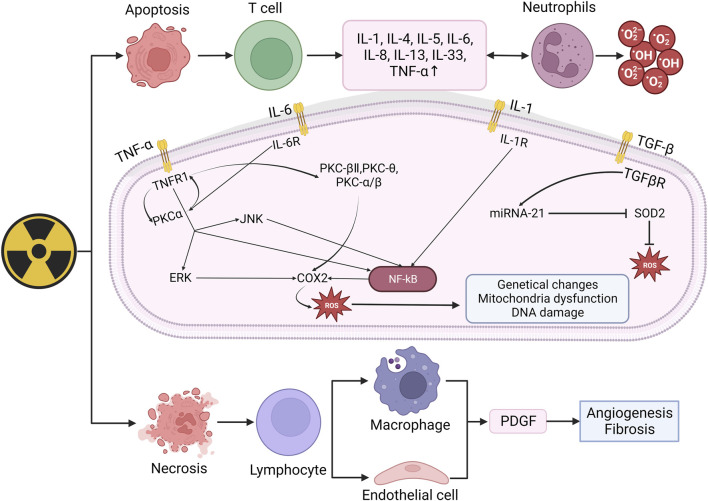
Mechanism of radiation-induced skin damage and OS. Radiation leads to apoptosis and necrosis and then upregulate the expression of IL-1, IL-4, IL-5, IL-6, IL-8, TNF-α. When combined with receptors respectively, these factors can activated NK-κB and eventually increase ROS. The increasing ROS may result in genetical changes, DNA damage and mitochondira dysfunction, thus lead to skin damage. (IL: Interleukin; TNF-α: Tumor necrosis factor: TGF-β, Transforming growth factor beta; PKC: Protein kinase C; ERK: Extracellular regulated protein kinases; JNK: c-Jun N-terminal kinase; NF-kB: Nuclear factor kB; SOD2: Superoxide dismutase 2; PDGF: Platelet-derived growth factor).

When irradiated, the early inflammatory response mainly manifests as the increase of pro-inflammatory cytokines (IL-1, IL-3, IL-5, IL-6, tumor necrosis factor TNF-α) and chemokines (IL-8) (Wei et al., 2018). These factors can activate neutrophils and produce ROS, thus lead to the damage of skin tissue and protective barriers. TNF-a, IL-6, and IL-1 are involved in the inflammatory response. When combined with receptors respectively, they can activate NK-κB through varies of ways and upregulate COX2, which eventually increase the number of ROS (Cheki et al., 2018). COX-2 can mediate the excessive production of arachidonic acids (such as prostaglandins and thromboxanes). When the levels of these AAs increase, tissues can become inflamed within hours and may last for weeks, occurring in various organs and tissues including the skin, such as the lungs, bones, and joints. Under normal physiological conditions, COX-2 is almost non-existent in most tissues of the body and only appears locally under inflammatory stimulation. Overexpression may lead to radiation resistance. Based on the above points, nonsteroidal anti-inflammatory drugs (NSAIDs) targeting COX-2 have good clinical potential for treating RISRs (Laube et al., 2016; Laube et al., 2013). The increasing ROS will result in genetical changes, DNA damage and mitochondria dysfunction (Yahyapour et al., 2018). Mitochondrial dysfunction typically included impaired respiratory chain function, structural abnormalities, depletion of cell ATP pool, disrupted cellular signaling and increased mitochondria-derived ROS (mtROS) generation (Dong et al., 2023). These mtROS will in turn induce OS and mitochondrial dysfunction. Besides, mitochondrial membrane integrity might be damaged during this process, releasing mitochondrial ligands or damage-associated molecular patterns (DAMPs), exacerbating the situation (Dela Cruz and Kang, 2018; Marchi et al., 2023). TGF-β also plays important role in radiation-induced skin damage, it has the function of wound healing, proliferation, differentiation of multiple cell types and synthesis of extracellular matrix proteins in inflammatory response of normal tissue. In the process of RISRs, TGF-β and platelet-derived growth factor (PDGF) can regulate fibroblast activity and promote the production of extracellular matrix proteins (Bentzen, 2006; Pohlers et al., 2009). Combined with its receptor, TGF-β upregulate the expression of miRNA-21, thus inhibit the superoxide dismutase 2 (SOD2), an important factor that eliminates ROS (Farhood et al., 2019) (Figure 2).

In conclusion, ionizing radiation induces the production and aggregation of ROS, leading to cell apoptosis or necrosis, thereby activating immune cells to produce a large number of inflammatory factors. When bound with corresponding receptors, these inflammatory factors activate the NF-κB pathway thus upregulating COX expression and ultimately produce more ROS, further leading to DNA damage and mitochondrial dysfunction. These signals and pathways stated above will not just function in the happening of radiation-induced skin damage as different types of RISRs are more like phased manifestations at different time points rather than individual reactions. As we know, when irradiation lasts for a period of time, acute RISRs would develop into chronic RISRs with radiation-induced skin fibrosis (RIF) being the most representative of which. In the next part, we will illustrate the mechanism of RIF.

### 2.2 Radiation-induced skin fibrosis

Radiation-induced skin fibrosis refers to a harmful chronic disease that appears weeks to years after radiation (Straub et al., 2015). When long-term ionizing radiation exposure causes damage to collagen fibers in the dermis of the skin, hardening of the skin and mucous membranes, RIF happens. It is a multicellular process involving the interaction of various cellular systems in skin tissue (Rodemann and Bamberg, 1995). The release of ROS, microvascular injury, recruitment of inflammatory cells, and activation of myofibroblasts all take part in the process of RIF (Wang et al., 2020), among which ROS play a significant role mainly through two pathways.

**FIGURE 3:**
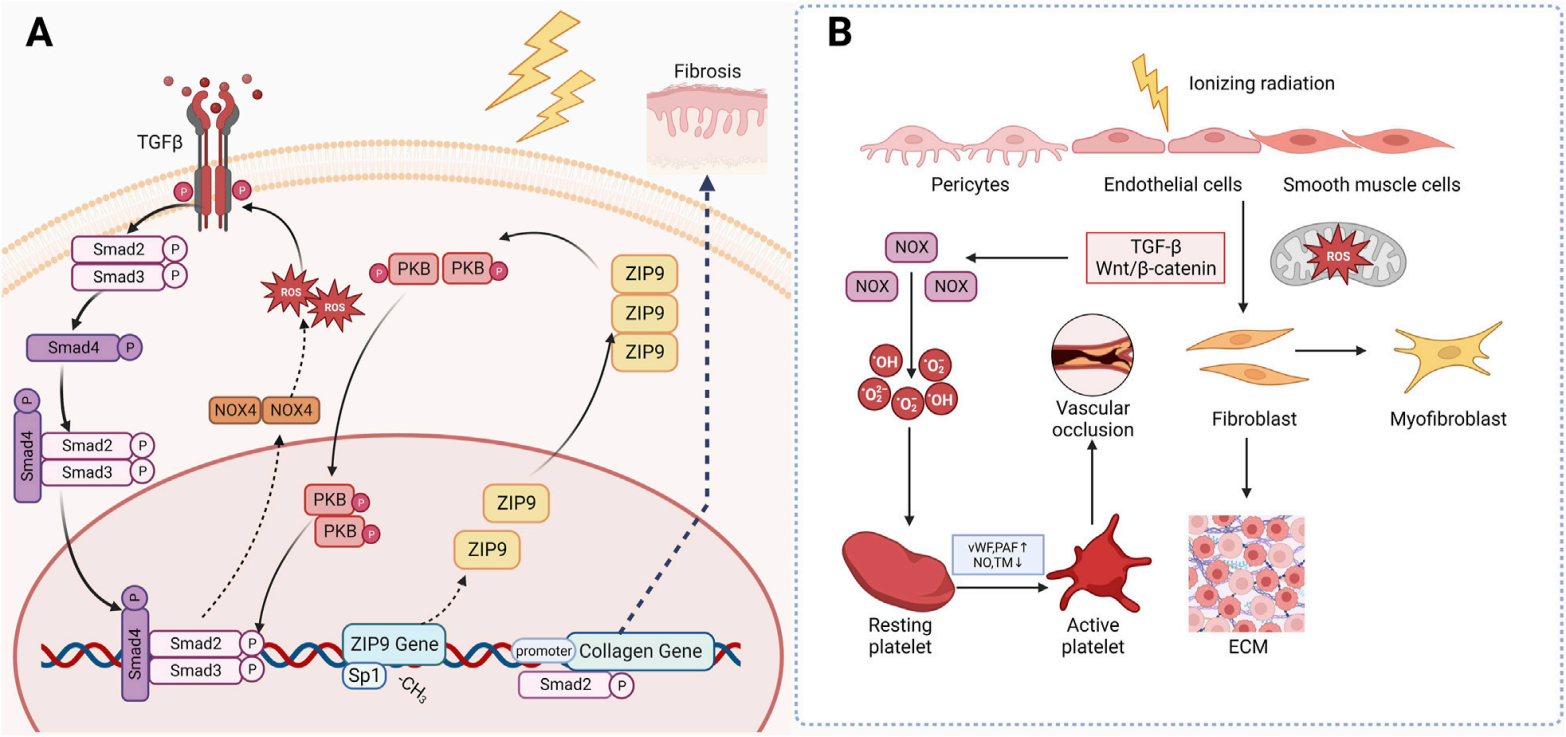
Mechanism of radiation-induced skin fibrosis and OS. **(A)**: Ionizing radiation induces the expression of ZIP9 and overexpression of ZIP9 activates the TGF- β/Smad signaling pathway and proceed the development of RIFs. **(B)**: Wnt/β-catenin is another classical profibrotic pathway that can induce the transformation of epithelial and endothelial cells into myofibroblasts. (TGF-β: Transforming growth factor beta; NOX4: NADPH oxidase 4; PKB: Protein Kinase B; ZIP9: Zrt-and Irt-like protein 9; vWF: Von Willebrand factor; PAF: Platelet activation factor; TM: Thrombomodulin).

One way is through a positive feedback regulation mechanism with TGF-β. When skin tissue is irradiated, monocytes, macrophages, fibroblasts, keratinocytes and many other cells will release IL-1, which will eventually increase the level of ROS in cells. ROS can activate TGF-β signal. The activated TGF-β then stimulates the Smad signaling cascade reaction, which in turn promotes ROS production by upregulating the transcription of NOX4 of NADPH, thereby a positive feedback regulation mechanism has been established between ROS and TGF-β (Richter et al., 2015; Derynck and Zhang, 2003). Ionizing radiation induces the demethylation of CpG dinucleotides in exon 1 of the Zrt-and Irt-like protein 9 (ZIP9), then recruit the transcription factor Sp 1 to promote ZIP9 expression. Overexpression of ZIP9 activates the TGF- β/Smad signaling pathway and proceed the development of RIFs (Qiu et al., 2020). Besides, human stromal cell-derived factor-1 α (SDF-1α) used to be thought as a key chemokine in the happening of diabetic nephropathy, but recently Zhang et al. found that it is also involved in radiation-induced skin damage and fibrosis as well (Cao et al., 2019). Briefly speaking, the SDF-1α/CXCR4 axis can promote the pro-fibrotic TGF-β/Smad signaling through PI3K-MAPK signaling cascade in human keratinocyte HaCaT cells and skin fibroblast WS1 cells, thus lead to skin fibrosis (Zhang et al., 2013). The other is through the disturbance of epigenetic modification. As stated above, when irradiated, the mitochondrial ROS will increase and change concentration of epigenetic metabolites, leading to modifications of the cellular epigenome (Yamamori et al., 2017) (Figure 3A).

Wnt/β-catenin is another classical profibrotic pathway. Inhibition of this pathway has been found to attenuate radiation-induced skin fibrosis through inhibiting the transformation of epithelial and endothelial cells into myofibroblasts (Lee et al., 2020). Microvascular injury may also be responsible for the occurrence of RIFs. After irradiation, the subendothelial Extracellular matrix (ECM) component is exposed to platelets, which promotes excessive secretion of von Willebrand factor (vWF), tissue factor and platelet activation factor (PAF), while reducing the production of NO, prostacyclin and transmembrane glycoprotein thrombomodulin (TM) and its fibrinolytic activity. These changes eventually trigger an antifibrinolytic-coagulation cascade effect, causing coagulation and vascular occlusion, accelerating the process of RIF (Soloviev et al., 2003) (Figure 3B).

In short, ionizing radiation-induced overexpression of ZIP9 activated the TGF-β/Smad signaling and upregulates NOX, thus leading to extensive production of ROS. These overproduced ROS then activates collagen genes resulting the occurrence of skin fibrosis. Besides, IR can activate platelet and fibroblast through Wnt/β-catenin and TGF-β, causing vascular occlusion and fibrosis. Based on what’ve been discussed above, it is not hard for us to find that OS and TGF-β play the plays a dominant role in the occurrence and development of RIF, leaving us the curiosity to go deeply into the potential of antioxidants and TGF-β targeted drugs in management of RISRs.

### 2.3 Radiation-induced skin carcinogenesis

Ultraviolet (UV) radiation contributes to the development of skin carcinogenesis through direct and indirect DNA damage, production of reactive oxygen species and local immunomodulation (Thompson and Kim, 2020). Cellular injury resulting from excessive ROS generation represents a consequence of interference with cellular membranes, proteins and DNA, changing the overall biological activities. As a result of these actions, oxidative products with mutagenic properties are formed, initiating the process of carcinogenesis within epidermal cells (Allegra et al., 2020; Ciążyńska et al., 2021). ROS are able to modify several pathways that are activated in tumors, including the activating protein-1 pathway, epidermal growth factor receptor, NF-κB, mitogen-activated protein kinase/extracellular signal-regulated kinase (MAPK/ERK), and p38 MAPK (Hammouda et al., 2020; van Hogerlinden et al., 1999; Loercher et al., 2004).

After irradiation, mutations accumulated in NOD-like receptor thermal protein domain associated protein1 (NLRP1) gene cause changes in the structures of domains, resulting in the formation of oligomers of NLRP1 and the activation of inflammasomes (Ciazynska et al., 2020), which can promote the production of caspase1, thus upregulating the expression of IL-1β and IL-18. These NLRP1-dependent production of IL-1β and IL-18 may contribute to the process of skin carcinogenesis (Ciazynska et al., 2020; King et al., 2019). It is also suspected that NLRP1 may involve in radiation-induced skin carcinogenesis by inhibiting the caspase-2/9-mediated apoptotic pathway (Zhai et al., 2017). Recently, NLPR3 has also been found to be a new approach to skin carcinogenesis (Wei et al., 2019b).

**FIGURE 4:**
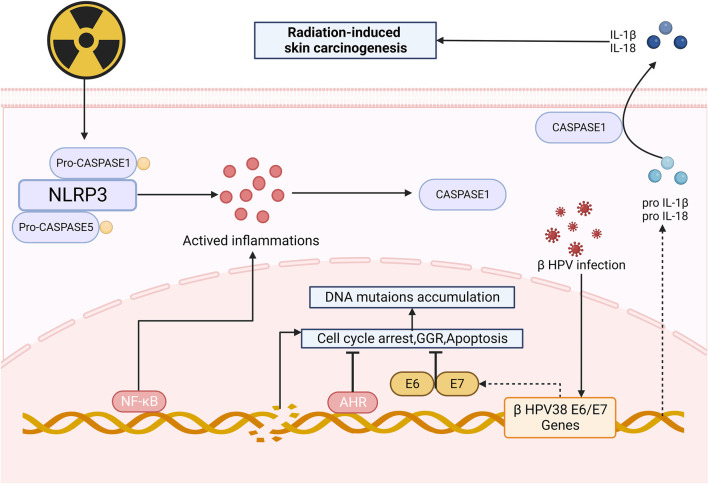
Mechanism of radiation-induced skin carcinogenesis and OS. After irradiation, the formation of oligomers of NLRP1 and the activation of inflammasomes can promote the production of caspase1, upregulating the expression of IL-1β and IL-18, thus leading to skin carcinogenesis. AHR and HPVs also take part in skin carcinogenesis, serving as facilitators. (NLRP3: Nucleotide-binding domain and leucine-rich-repeatcontaining family pyrin 3; AHR: radiation aryl hydrocarbon receptor; IL-1β: Interleukin-1β; GGR: Global genome repair).

Radiation aryl hydrocarbon receptor (AHR) signaling is also responsible to radiation-induced skin carcinogenesis. AHR accelerates the process of RISRs as a sensitizer for it increases after irradiation and expression of mRNA and protein of both cytochrome P450 1A1 and cytochrome P450 1B1 in the epidermis are enhanced, upregulating the bioactivation of environmental pollutants, thereby making human skin more susceptible to radiation-induced skin cancers or dermatitis (Katiyar et al., 2000). The global genome repair (GGR) and apoptosis in epidermal keratinocytes (KC) are repressed (Pollet et al., 2018) after exposure to UV and sensitivity of cells to PAH-mediated DNA adduct formation increase (Nair et al., 2009), ultimately contributes to skin photo carcinogenesis. Octinoxate, an inhibitor of cytochrome 1A1 (CYP1A1) and cytochrome 1B1 (CYP1B1), can elevate CYP1A1 and CYP1B1 mRNA levels in mouse and thus modulate AHR signaling, indicating that it might exhibit protective effect on human skin. Topical application of BDDI (E/Z-2-benzylidene-5,6-dimethoxy-3,3-dimethylindan-1-one), a type of AHR antagonist, can significantly reduce the UVB-induced expression of carcinogenic genes (Tigges et al., 2014). Beta HPVs are another risk factors of skin carcinogenesis since it can promote proliferation and circumvent cellular stresses via the E6 and E7 oncoproteins when being irradiated (Tommasino, 2019) (Figure 4).

Carcinogenesis is a complicated process that involves multiple factors, pathways and so on. In this context, we briefly summarize the mechanism related to HPV infection and NLRP3 inflammasome. Although the frequency of radiation-induced skin carcinogenesis is not that high as other types of RISRs, its negative impact on patient survival and QoL is profound and significant, which is worthy of our attention. Unfortunately, due to its rarity and the complexity of its mechanisms, there are not many existing research results, further explorations still need to be done.

### 2.4 Radiation-induced skin aging

Aging is a natural intrinsic process associated with the loss of fibrous tissue, slower cell turnover and reduction in immune system competence. Many factors contribute to akin aging, among which the most significant one is ultraviolet radiation. Skin aging events are initiated and often propagated by oxidation events though invisible to naked eyes (D’Arino et al., 2023; Kammeyer and Luiten, 2015). The effects of skin damage caused by UV radiation produce injuries that are mainly invisible to the naked eye (Silva Acosta et al., 2021). Both intrinsic (proliferative exhaustion and telomere shortening) and extrinsic stresses as well as the activation of oncogenes can lead to cellular senescence (Lee et al., 2021). How do extrinsic stresses cause skin ageing? One of the core mechanisms mediating skin aging lie in the OS induced by the accumulation of ROS, which can lead to lipid, protein, nucleic acid damage, thus leading to the occurrence of cellular senescence (Gu et al., 2020). Photoaging is mainly caused by repeated exposure to UVA radiation. To be more specific, the increased ROS levels will result in DNA damage, collagen degradation and release of inflammatory corpuscles. Besides, ROS can cause destructive OS, activating the arachidonic acid pathway and mediating inflammatory responses (Gu et al., 2020).

Radiation induced DNA damage response (DDR) leads to stimulating p38/MAPK/PKC/NF-κB, contributing to increased intracellular ROS, thus activating the Cyclin-dependent kinase inhibitor 2 A (CDKN2 A) locus, producing p16 INK4A (p16) and p19 INK4D (ARF). p16 INK4A can activate the retinoblastoma protein (pRb) tumor suppressor, then blocks certain proliferative genes by heterochromatinization. Ultimately durable cell-cycle arrest was induced (Nguyen et al., 2018). Besides, UV radiation induced ROS can lead to the phosphorylation and subsequent activation of c-Jun N-terminal kinases. These activated kinases then activate the c-Jun and c-Fos components of the transcription factor activator protein-1 (AP-1), increasing the expression of elastolytic matrixmetallo proteinase (MMPs) (Fitsiou et al., 2021). Saguie et al. found that after irradiation, expression of inflammatory cells, collagenolytic and MMPs increases while elastin expression decreases, thus leading to photoaging (Saguie et al., 2021). MMPs are highly expressed in UV-induced senescent KCs in culture (e.g., MMP-1) and in the epidermis of irradiated human skin samples (e.g., MMP-1, MMP-3, and MMP-9) (Dong et al., 2008; Quan et al., 2009). Proinflammatory cytokines such as TNF-α, IL-1α, IL-1β, and IL-6 also increase in UV-induced senescent KCs (Bashir et al., 2009).

**FIGURE 5:**
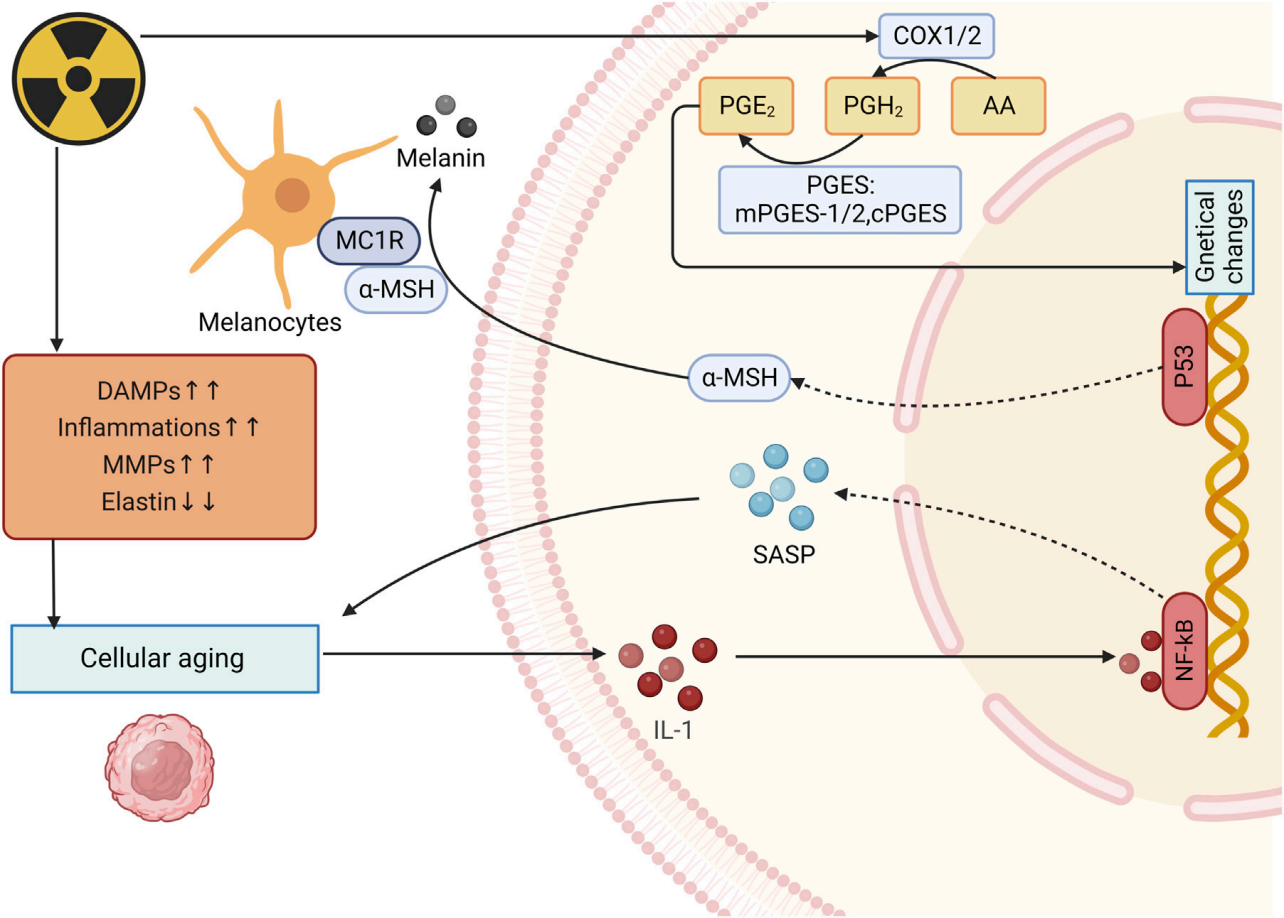
Mechanism of radiation-induced skin aging and OS. After irradiation, expression of inflammatory cells, collagenolytic and elastolytic matrixmetallo proteinase (MMPs) increases while elastin expression decreases, thus leading to photoaging and increase of IL-1expression. IL-1 upregulates SASP expression and enhance the photoaging. UV radiation-induced DNA damage activates p53 and eventully activates melanogenesis. (DAMPs: Damage-associated molecular patterns; MMPs: Matrixmetallo proteinases; PGE2: prostaglandin E2; SASP: Senescence-associated secretory phenotype; α-MSH: α-melanocyte-stimulating hormone).

Skin pigmentation is one of the main symptoms of skin aging, which is mainly caused by intracellular melanin deposition. UV radiation-induced DNA damage in keratinocytes activates p53 which initiates the transcription of proopiomelanocortin and subsequently peptides including α-melanocyte-stimulating hormone (α-MSH) are formed. These peptides then combined with MC1R on melanocytes to induce microphthalmia transcription factor (MITF), activating melanogenesis (Lee et al., 2021) (Figure 5).

Many hormone-like mediators are involved in the process of photoaging. For example, level of prostaglandin E2 (PGE-2) increases quickly when skin is exposed to UVR, causing changes in genetical changes and eventually lead to cell dysfunction (Fuller, 2019) (Figure 5). Biskanaki et al. (2021) found that skin collagen type I (COL I) decreases during aging, even more in photoaging, which indicates that radiation can reduce skin COL I expression and contributes to negative impacts on the skin. Besides, when photoaging occurs, IL-1 expression is upregulated and it can bind to IL-1 receptor/Toll-like receptors to activate NF-κB, thereby increasing the expression of SASP (Gu et al., 2020; Chang et al., 2002). Furthermore, senescence-associated secretory phenotype (SASP) can promote the malignant transformation of adjacent recipient cells, leading to the occur of inflammation, diseases and cells aging (Campisi et al., 2011).

The release of SASP, α-MSH and PGE-2 has a lot to do with the happening of radiation-induced skin aging. The mechanism of radiation-induced skin aging shares many similarities with skin cancer, because skin aging can be seen as a precursor to skin cancer despite the fact that not all skin aging ultimately develops into skin cancer, the relationship between these two is still inseparable. Deeply studying the mechanism of radiation induced skin aging can help identify treatment for managing RISRs and improve patient QoL, besides, provide insights into the pathogenesis of skin cancer.

## 3 Antioxidant protection

### 3.1 Antioxidant drugs

Early in 1963, people have realized the effectiveness of corticosteroids in healing RISRs (Jackson, 1963). Corticosteroids have the abilities of anti-inflammatory, reversing the skin related decline in QoL and prohibiting radiation-induced cytokine proliferation (Schmuth et al., 2002a; HARUNA et al., 2017; Hymes et al., 2006), making it exhibit protective effect on skin. Topical usage of corticosteroids has proven be effective in reducing eczema peeling, frequency of serious skin toxicity and delaying the occurrence of grade 1 to 3 dermatitis (HARUNA et al., 2017). However, there is still controversy over whether routine steroid local therapy should be given to patients receiving radiation therapy and its optimal timing of application. The results of three related trials did not show that steroid local therapy can prevent the occurrence of RISR (Glees et al., 1979; Miller et al., 2011; Løkkevik et al., 1996; Yokota et al., 2021; Zenda et al., 2018). Only a small trial of 20 people observed effective differences, indicating that corticosteroids may not be an effective means of preventing RISR (Halnan, 1962). A large clinical trial showed that topical application of 0.1% mometasone furoate cream was not superior to placebo in reducing the maximum RISR level or RISR level at 2 weeks after treatment, while two small trials showed that steroid topical treatment was effective in reducing RISR levels (Schmuth et al., 2002b). Nonsteroidal drugs such as hyaluronic acid (HA) and zanzanamine lotion also have some therapeutic effects. HA is a type of carbohydrate polymer. Topical applied HA creams has been suggested to be protective against ROS damage caused by radiation in animal researches. Whether it can help with alleviating human skin injuries remain unclear, for one study found that it reduced the incidence of high-grade RD while the other witnessed more severe dermatitis. Whether it can be used in patients who received RT still need further researches. Trolamine emulsion have similar effect. Serving as macrophage cell stimulators, they are able to remove necrotic tissue, promote fibroblast proliferation, reduce vascular alterations, restore CD34 expression, promote epithelial cell proliferation, and decrease IL-1 expression and collagen secretion (Hymes et al., 2006). Abbas et al. demonstrated that when used in patients who accepted radiation therapy, Trolamine emulsion indeed significantly reduced the intensity of acute dermatitis by its non-steroidal anti-inflammatory properties and ability to recruit macrophages to the wound bed and promote the production of granulation tissue (Abbas and Bensadoun, 2012). 2-Methoxyestradio may also be a potential therapeutic agent for RISRs as it can repair the damaged vessels on the irradiated dermal skin, inhibit endothelial HIF-1 alpha expression and fibrotic changes and accumulate DNA damage in irradiated human dermal microvascular endothelial cells (Kim et al., 2022) (Tables 1, 2).

**TABLE 1:** Antioxidant protections of RISRs.

Methods	Mechanism	Reference
Antioxidant Drugs		
Corticosteroids	Prohibit radiation-induced cytokine proliferation	Haruna et al. (2017)
Trolamine emulsionis	Act as macrophage cell stimulators	Abbas and Bensadoun (2012)
Sucralfate	Act as a mechanical barrier with antibacterial, anti-inflammatory function	Le et al. (2024)
Heme oxygenase-1(HO-1)	Share antioxidant and antiapoptotic properties	Zhang et al. (2012)
Injections of MnSOD	Inhibit of Ferroptosis	Wang et al. (2024)
Antioxidant Materials		
3M barrier film	Reduce friction and irritation	Shaw et al. (2015)
HBOT	Inhibit the TGF-β pathway	Spiegelberg et al. (2014); Pandey et al. (2022)
Triethanolamine cream	Facilitate microcirculation	Yang et al. (2020)
Antioxidant Cytokines and Vesicles		
Sulforaphane (SFN)	Upregulate the expression and function of Nrf2	Wei et al. (2021)
Interleukins (ILs)	Reduce the retention of cutaneous dendritic cells	Gerber et al. (2015)
T-cell protein tyrosine phosphatase	Inhibit Smad2 nuclear translocation	Morales et al. (2019)
Superoxide dismutase (SOD)	Antioxidants that clear ROS	Choung et al. (2004); Pudlarz et al. (2020); Yu et al. (2019)
Aryl hydrocarbon receptor (AHR)	Reduce the UVB-induced expression of fibrosis gene	Tigges et al. (2014)

**TABLE 2:** Clinical trials of antioxidant protection.

Clinical trials	Registration number	Person in charge	Cases	Protocol (study vs. control)	Results
CSMed wound dressing for radiation dermatitis	NCT06001463	Lee et al. (Lee et al., 2024)	30	Half of the irradiated area was covered with CSMed^®^ and the other half was under routine treatment.	CSMed^®^ dressed area had significant lower RTOG score during and after RT than undressed area.
KeraStat(R) cream for radiation dermatitis	NCT03559218	Karen et al. (Winkfield et al., 2024)	25	Study: KeraStat cream for twice daily application; control: standard of care.	The rate of RTOG Grade 2 RD was lower in the study group (30.8%) compared to the control group (54.5%, *P* = 0.408).
Silver-nylon dressing (Silverlon^®^) to reduce radiation dermatitis	NCT04238728	Julie et al.	31	Silver nylon dressing will be applied daily	3.6% (1/28) patients reported severe adverse skin event and mean healthcare professional assessment scale (HPAS) was 6.31 (95% CL = 5.47–6.94).
Photobiomodulation and photodynamic therapy for the treatment of oral mucositis	—	Erika et al. (Pires Marques et al., 2020)	56	Control: low-level laser applied in the oral cavity; study: additionally gave photosensitizing mouthwash and blue LED.	Both groups observed significant reductions in OM grade (p < 0.0001), while study group resulted in a shorter time to resolution of lesions (p = 0.0005).
Dakin’s solution in preventing radiation dermatitis	NCT02203565	Kathleen et al. (Hejazi et al., 2016)	20	Tropically applied Dakin’s solution daily during RT for up to 6 weeks.	15% (3/20) patients experienced grade 3/4 radiation dermatitis.
Oral curcumin for radiation dermatitis	NCT01246973	Julie et al. (Ryan Wolf et al., 2018)	68	Study: curcumin C3 complex (2.0 g) taken orally 3 times/day control: Placebo.	Curcumin did not reduce radiation dermatitis severity (B (95% CI) = 0.044 (- 0.101, 0.188), p = 0.552).
Effect of curcumin supplementation during radiotherapy on oxidative status	NCT01917890	Jalal et al. (Ertekin et al., 2004)	40	Study: curcumin capsules (BCM95, Biocurcumin); control: placebos (roasted rice flour of 500 mg).	No significant differences were observed between the 2 groups regarding treatment outcomes.
Oral glutamine on radiation dermatitis	NCT03015077	Huang et al. (Huang et al., 2019)	71	Study: oral glutamine (5 g glutamine and 10 g maltodextrin); control: placebo (15 g maltodextrin).	No difference was found in the incidence and severity of neck dermatitis between the two arms.
Bacterial decolonization for prevention of radiation dermatitis	NCT03883828	Yana et al. (Kost et al., 2023)	80	Study: intranasal mupirocin ointment twice daily and chlorhexidine body cleanser; control: standard of care.	The mean ARD grade was significantly lower for BD group compared with standard of care (1.2 vs. 1.6) (*P* = 0.02).
Topical superoxide dismutase in posttreatment fibrosis	—	Kelly et al. (Landeen et al., 2018)	68	Study: 280 IU/g SOD to the area of fibrosis twice daily control: placebo lotion.	46.4% of study group improved by 1 or more ranks on the CTCAE v4.0 scale, compared to 43.3% of control group.
Oral zinc sulphate on anti-oxidant enzyme activities	—	Ertekin et al. (Ertekin et al., 2004)	30	Study: zinc sulphate capsules (including 50 mg zinc) three times a day; control: placebo.	No difference was detected in any final measurement activities of erythrocyte anti-oxidant enzymes in the direct comparison between two groups.
Phase 3 randomized trial of topical steroid versus placebo for prevention of radiation dermatitis		Tomoya et al. (Yokota et al., 2021)	211	Study: topical steroid when grade 1radiation dermatitis was observed or the total radiation dose reached 30 Gy control: placebo	

Ascorbic acid, also known as vitamin C, possesses powerful antioxidant and free radical scavenging qualities. Unfortunately, the benefit of topical ascorbic acid in human being has not been demonstrated yet (Rosenthal et al., 2019), but it seems to have a promising future. Vitamin E also has similar antioxidant properties. Delinasios et al. (2018) found that the use of vitamin E before or after RT can reduce the formation of oxidative purine and cyclobutane pyrimidine dimers (CPD), which supports the use of vitamin E to reduce side effects in patients receiving RT. The effects of antioxidants such as vitamin C and vitamin E are very extensive and powerful. In the past, they often acted through oral administration. They have the characteristic of reducing the formation of CPD, which may still enable them to have effective preventive and therapeutic effects. When added as additives to topical medications such as sun protection.

In recent news, ferroptosis, a form of cell death caused by ROS overload, has been found to be involved in the occurrence of RISRs. Radiation exposure could induce the accumulation of lipid peroxides in human skin keratinocytes, resulting in intracellular Fe2+ overload by regulating the levels of iron exporter ferroportin (FPN) (Su et al., 2022). According to this discovery, local Multiple-site injections of a plasmid harboring human MnSOD may exert protective in patients following RT by inhibiting ferroptosis (Wang et al., 2024). Transdermal delivery of the iron chelator deferoxamine (DFO) can improve tissue perfusion and mitigates chronic RIF (Shen et al., 2020; Lavin et al., 2021). Heme oxygenase-1 (HO-1), the rate-limiting enzyme in heme catabolism, has been reported to have potential antioxidant and antiapoptotic properties, making them potential to be used in alleviating RISRs (Zhang et al., 2012). Similarly, topically applied fibronectin has been found to significantly improve wound healing in irradiated skin due to its effect of decreasing inflammatory infiltrate and increasing angiogenesis (Johnson et al., 2017).

These drugs mentioned above all have antioxidant effects or anti-inflammatory to varying degrees, but currently, most of the more widely used traditional drugs can only alleviate symptoms. New drugs that can truly cause inhibit RISRs from the beginning are either not widely available or are still in the experimental stage. As we gradually establish a deeper understanding of the mechanism of radiation damage, we have found that more and more cytokines, signaling pathways, and other factors are involved. It is possible to achieve the goal of inhibiting RISRs by developing drugs targeting these factors in the future.

### 3.2 Antioxidant materials

3M barrier can reduce friction and irritation, especially in skin folds and thin areas. A completed observational clinical study from the United States (NCT03546803) showed that the 3M Avilon advanced skin protector can to some extent prevent radiation dermatitis in cancer patients, indicating that using the 3M barrier at the beginning of radiotherapy is a possible method for preventing RISRs (Table 1).

A non-randomized study has confirmed that the RISR was significantly lower in the group receiving the sucralfate gel, highlighting the benefit of sucralfate humid gel in alleviating RISRs. Sucralfate humid gel is the colloidal physical form of the anti-ulcer drug sucralfate, which can form a protective layer, increase bicarbonate production, exhibit anti-peptic effects, and promote tissue growth, regeneration and repair (Le et al., 2024). Similarly, triethanolamine cream, a compound preparation with good hydration, has the ability to drain and cleans the skin, reduce patients’ skin dryness, decrease body inflammation and edema response, facilitate patients’ body microcirculation and enhance skin tolerance, making it a useful protection in management of RISRs (Yang et al., 2020).

Evoskin and Trixiera are two topical moisturizing and repairing creams, and their effectiveness was compared in a completed clinical trial by a French research team (NCT02334345). The research team included women with cancer who received 50 Gy breast radiation therapy in a prospective randomized trial. Patients used Evoskin in half of the irradiated breasts and Triesiera in the other half, and the results showed that Evoskin was more effective than Trixiera. This indicates that the use of Evoskin is highly recommended for patients undergoing radiation therapy. Similarly, a randomized phase III study (NCT00876642) evaluated the efficacy of aloe vera cream in preventing grade 2 or higher acute radiation dermatitis during postoperative radiotherapy for cancer. However, although many pilot studies have shown that aloe gel has antioxidant and anti-inflammatory properties, it may not be effective for the prevention or treatment of radiation adverse reactions in breast cancer patients, and may only be effective for some patients with cumulative radiation doses greater than 27 Gy and acute radiation proctitis (Farrugia et al., 2019).

Other potential ways of improving RISRs resulted from RT include hyperbaric oxygen therapy (HBOT), surgery, transplantation of endothelial cells and so on. HBOT function mainly through suppressing the TGF-β expression (Spiegelberg et al., 2014). Safra et al. suggested that HBOT be a safe and effective countermeasure to radiation-induced late side-effects (Safra et al., 2008) and particularly can also improve skin elasticity in patients with RIF (Pandey et al., 2022). The future of transplantation of stromal vascular fraction (SVF) in addressing RISRs is promising (Yu et al., 2021). A recruited clinical study conducted by a team from Boston, United States is investigating how to use epidermal skin grafts to improve radiation wound healing (NCT04560803). They mainly use CelluTome, an epidermal transplant system, to form epidermal vesicles to cover the wound, and evaluate the efficacy of epidermal grafts collected using the CelluTome device.

Local application of materials that can achieve antioxidant effects can also help alleviate RISR, such as using 3M barriers, HBOT, and various cell transplants. These materials mainly exert therapeutic effects by establishing barriers, clearing ROS, and inhibiting inflammatory factors. With the continuous development of radiation therapy technology, serious skin reactions have become very rare. Therefore, although studies have shown that cell transplantation can alleviate severe RISR and its clinical application is limited, this does not mean that this research direction is unnecessary. Because RISR shares common mechanisms with other skin diseases, which means that the up-to-date treatment of RISR may also provide ideas for the treatment of other skin diseases, making it a very promising research direction.

### 3.3 Antioxidant cytokines and vesicles

Interleukins (ILs) are closely involved in multiple steps in the occurrence and development of RISRs. Among which IL-12 has been recognized as a potential mitigator of RISRs by an American team since it can reduce the retention of cutaneous dendritic cells, slow down transepidermal water loss and minimize burn size (Gerber et al., 2015). As stated above, NLRP1 and NLRP3 activation plays a significant role in the happening of RISRs by upregulating the expression and function of Nrf2 and causing OS. Sulforaphane (SFN) may be able to prevent and alleviate RISRs by inhibiting these actions (Wei et al., 2021) (Table 1).

Tetrahydrobiopterin (BH4), also known as Sapropterin, is an important cofactor of nitric oxide synthase (NOS), and guanosine triphosphate hydrolase 1 (GCH1) is a key enzyme in the synthesis of BH4. Overexpression of GCH1 can restore BH4 levels and NO products in irradiated skin cells, reverse and inhibit NOS uncoupling caused by ionizing radiation, thereby eliminating ROS induced by ionizing radiation and reducing DNA damage (Spencer et al., 2005; Schallreuter et al., 1994a; Schallreuter et al., 1994b; Feng et al., 2021; Kraft et al., 2020). In animal experiments, direct subcutaneous injection of BH4 can not only reduce the severity of acute radiation skin injury, but also promote skin repair, reduce the occurrence of radiation skin fibrosis, and maintain normal physiological functions of the skin. In order to explore the efficacy and safety of tetrahydrobiopterin in the treatment of human radiation dermatitis, a related clinical study is being recruited (NCT05114226). The main observation endpoint is the incidence rate of acute radiation dermatitis.

Superoxide dismutase (SOD) is an antioxidant enzyme with multiple metal cofactors that can specifically clear ROS, which plays an important role in a variety of ultraviolet-induced lesions. Therefore, SOD has the anti-ultraviolet radiation effect (Chen et al., 2023; Choung et al., 2004; Pudlarz et al., 2020). The total flavonoids of boxthorn leaves improve resistance to RISRs by regulating SOD level in the skin of mice (Yu et al., 2019). A clinical trial has seen witnessed regression of fibrosis through 2-month follow-up in 34 patients treated with sixintramuscular injections of SOD over a 3-week period (Delanian et al., 1994), directly confirmed the protective role in anti-RISRs. SOD can been regarded as the most representing example of antioxidants functioning in skin protection from radiation, and presents us with faith in exploring more antioxidants to address the side effect of RT.

Antioxidant cytokines and vesicles, whether artificial or natural, can help alleviate RISRs by clearing ROS and inhibiting inflammatory cells. At the same time, mitochondrial dysfunction is one of the important reasons for ROS aggregation, making many mitochondrial targeted antioxidant cytokines a prospective countermeasure to RISRs. Many of these factors are extracted from herbs, indicating that herbs have great potential for RISRs management. However, we still need to explore the specific components in herbs that actually exert therapeutic effects for it can not only help us understand the mechanism of RISRs, but also avoid unnecessary loads caused by other impurities in herbs as much as possible.

## 4 Conclusion

In this review we summarize the mechanism of each type of RISRs and briefly conclude some countermeasures. Although traditional treatments like topical steroids, creams, and ointments have been widely used in clinics to alleviate RISRs, they are only symptomatic treatment with unsatisfactory effect. We still need more clinical trials and studies to validate the effectiveness and safety of innovative treatments like antioxidants to prevent the happening of RISRs from the root cause, and to achieve that, further studies focused on the mechanisms of RISRs and corresponding targeting drugs are urgently demanding.

## Glossary

AHR: Radiation aryl hydrocarbon receptor
ARF: p19 INK4D
BDDI: E/Z-2-benzylidene-5,6-dimethoxy-3,3-dimethylindan-1-one
CCE: C. costata extract
CDKN2 A: Cyclin-dependent kinase inhibitor 2 A
COL I: Collagen type I
CTS: Cryptotanshinone
DAMPs: Damage-associated molecular patterns
ECM: Extracellular matrix
ERK: Extracellular regulated protein kinases
FPN: Ferroportin
GGR: Global genome repair
HA: Hyaluronic acid
HBOT: Hyperbaric oxygen therapy
HO-1: Heme oxygenase-1
IL: Interleukin
JNK: c-Jun N-terminal kinase
KC: Keratinocytes
MITF: Microphthalmia transcription factor
MMP: Matrixmetallo proteinase
MSCs: Mesenchymal stem cells
mtROS: mitochondria-derived ROS
NADP: Nicotinamide-adenine dinucleotide phosphate
NF-kB: nuclear factor kB
NLRP3: Nucleotide-binding domain and leucine-rich-repeatcontaining family pyrin 3
NOX4: NADPH oxidase 4
OS: Oxidative stress
PAF: Platelet activation factor
PDGF: Platelet-derived growth factor
PGE-2: Prostaglandin E2
PKB: Protein Kinase B
PKC: Protein kinase C
pRb: retinoblastoma protein
QoL: Quality of life
RA: Rosmarinic acid
RIF: Radiation-induced skin fibrosis
RISRs: Radiation-induced skin reactions
RNS: Reactive nitrogen species
ROS: Reactive oxygen species
SASP: Senescence-associated secretory phenotype
SDF-1α: Human stromal cell-derived factor-1 α
SFN: Sulforaphane
SOD: Superoxide dismutase
SOD2: superoxide dismutase 2
SVP: Stromal vascular fraction
TC-PTP: T-cell protein tyrosine phosphatase
TGF-β: Transforming growth factor beta
TM: Thrombomodulin
UV: Ultraviolet
vWF: von Willebrand factor
ZIP9: Zrt-and Irt-like protein 9
8-OHdG: 8-hydroxydeoxyguanosine
α-MSH: α-melanocyte-stimulating hormone.
